# Supplementary material for: Candida parapsilosis Cell Wall Proteome Characterization and Effectiveness against Hematogenously Disseminated Candidiasis in a Murine Model
Source: Vaccines (Basel). 2023 Mar 16;11(3):674. doi: 10.3390/vaccines11030674 (PMC10059874; doi:10.3390/vaccines11030674)
Supplement: Supplementary file 1 [file vaccines-11-00674-s001.zip › vaccines-2268243-supplementary.pdf]

Supplementary File S1: Result of VITEK 2 analysis for the colonies after CFU plate counting

CMJAH

Laboratory Report

bioMérieux Customer:
System #: 3809
Isolate: XG-1 (Approved)
Card Type: YST Bar Code: 2432155503725447 Testing Instrument: 000015F2342D (CMJAH)
Setup Technologist: thulile Sibiyatthulile)

Printed by: Labadmin
Bench: mycology

Bionumber: 4112144645111731
Organism Quantity:

Selected Organism: Candida parapsilosis

**Comments:**	

**Identification Information**	**Card:** YST		**Lot Number:** 2432155503		**Expires:** Oct 19, 2023 13:00 CDT	
	**Status:** Final		**Analysis Time:** 18.02 hours		**Completed:** Oct 29, 2022 10:41 CDT	
**Organism Origin**	VITEK 2					
**Selected Organism**	90% Probability		**Candida parapsilosis**			
	Bionumber: 4112144645111731				Confidence: Good identification	

Analysis Organisms and Tests to Separate:

Analysis Messages:
See product information for additional information.

Contraindicating Typical Biopattern(s)
Candida parapsilosis dXYLa(85),NAGa(78),MAdGa(99),dGATa(88),GLYLa(88).

Biochemical Details

3	LysA	-	4	IMLTa	-	5	LeuA	+	7	ARG	-	10	ERYa	-	12	GLYLa	-
13	TyrA	-	14	BNAG	-	15	ARBa	-	18	AMYa	-	19	dGALa	+	20	GLNa	-
21	dGLUa	+	23	LACa	-	24	MAdGa	-	26	dCELa	-	27	GGT	-	28	dMALa	+
29	dRAFa	-	30	NAGA1	-	32	dMNEa	+	33	dMELa	-	34	dMLZa	+	38	ISBEa	(+)
39	IRHAa	-	40	XLTa	-	42	dSORa	+	44	SACa	-	45	URE	-	46	AGLU	+
47	dTURa	+	48	dTREa	-	49	NO3a	-	51	IARAa	(+)	52	dGATa	-	53	ESC	-
54	lGLTa	+	55	dXYLa	-	56	LATa	-	58	ACEa	-	59	CITa	+	60	GRTas	(+)
61	lPROa	-	62	2KGa	-	63	NAGa	-	64	dGNTa	+						

Installed VITEK 2 Systems Version: 9.02
MIC Interpretation Guideline:
AES Parameter Set Name:

Therapeutic Interpretation Guideline:
AES Parameter Last Modified:

bioMerieux Customer:
System #: 3809
Isolate: SGS-1 (Approved)
Card Type: YST Bar Code: 2432069503657210 Testing Instrument: 000015F2342D (CMJAH)
Setup Technologist: thulile Sibiya(thulile)

Printed by: Labadmin
Bench: mycology

Bionumber: 4112544645311771
Organism Quantity:

Selected Organism: Candida parapsilosis

Action	Name (User ID)	Date/Time	Comment
